# Supplementary material for: Effects of Timing of Acute and Consecutive Catechin Ingestion on Postprandial Glucose Metabolism in Mice and Humans
Source: Nutrients. 2020 Feb 21;12(2):565. doi: 10.3390/nu12020565 (PMC7071372; doi:10.3390/nu12020565)
Supplement: Supplementary file 1 [file nutrients-12-00565-s001.pptx]

## Slide 1
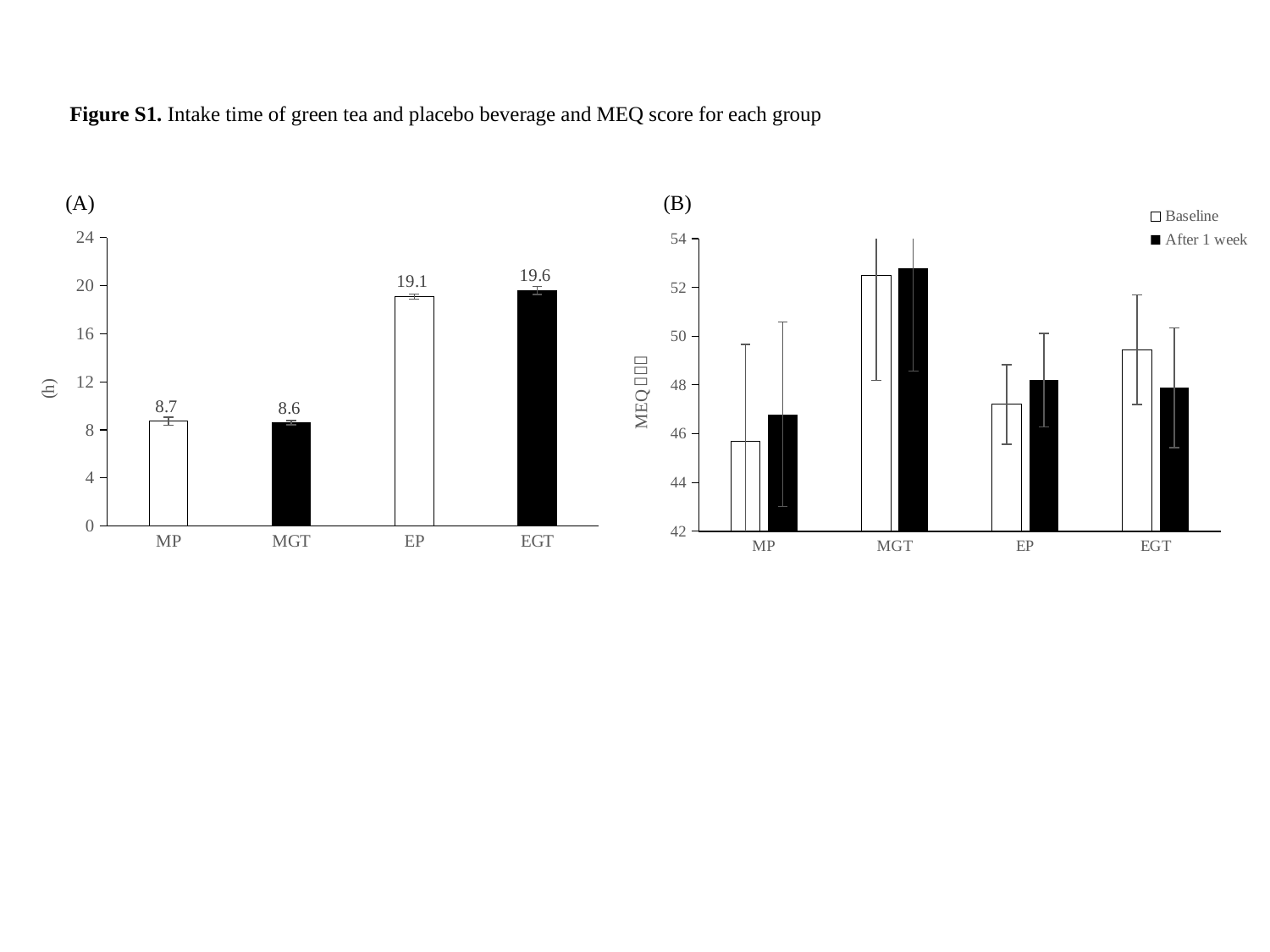

Figure S1. Intake time of green tea and placebo beverage and MEQ score for each group
(B)
(A)
### Chart
| Category | |
|---|---|
| MP | 8.729166666666668 |
| MGT | 8.586111111111112 |
| EP | 19.09104938271605 |
| EGT | 19.584876543209877 |
### Chart
| Category | Baseline | After 1 week |
|---|---|---|
| MP | 45.7 | 46.8 |
| MGT | 52.5 | 52.8 |
| EP | 47.2 | 48.2 |
| EGT | 49.44444444444444 | 47.888888888888886 |

## Slide 2
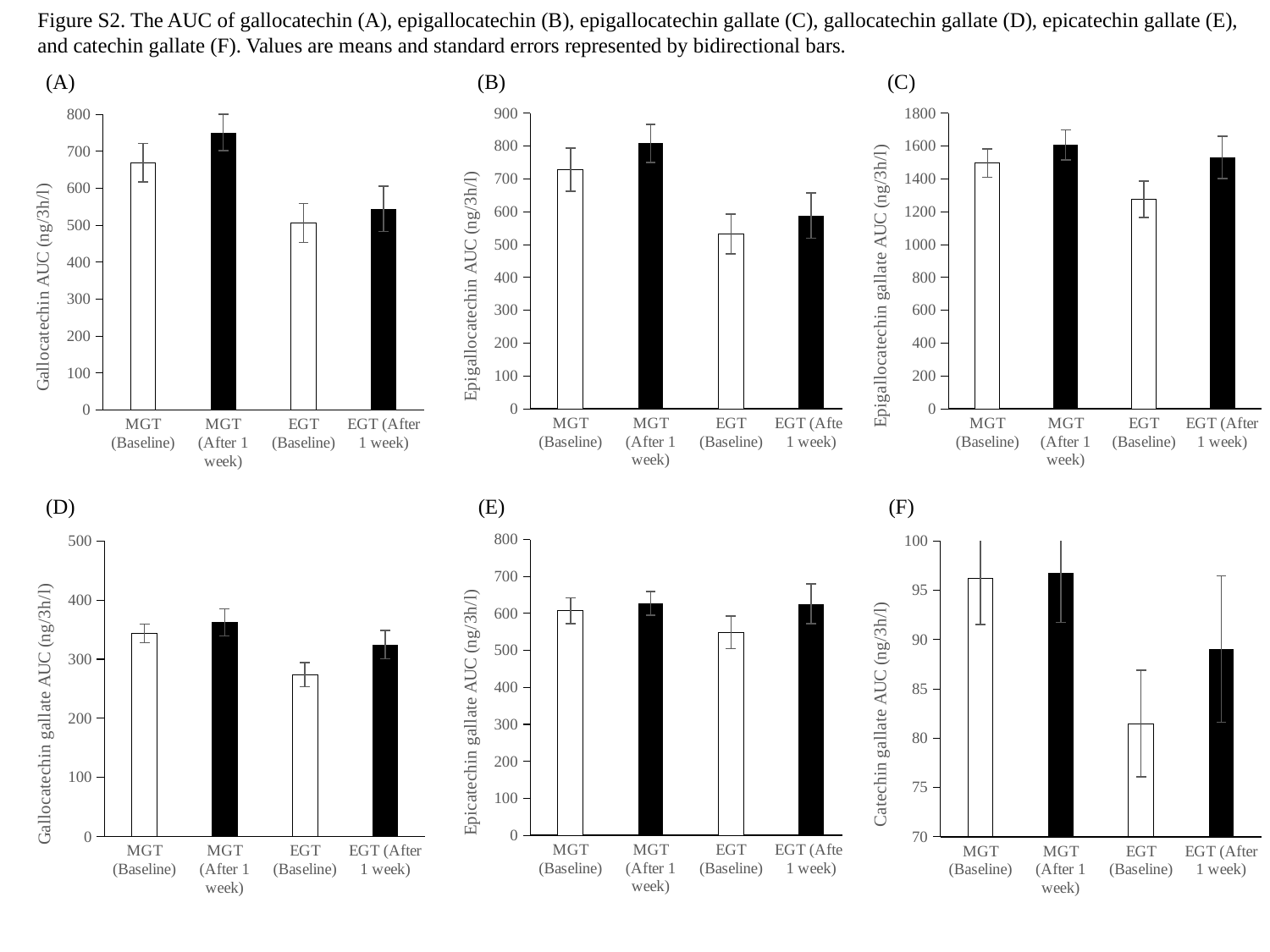

Figure S2. The AUC of gallocatechin (A), epigallocatechin (B), epigallocatechin gallate (C), gallocatechin gallate (D), epicatechin gallate (E), and catechin gallate (F). Values are means and standard errors represented by bidirectional bars.
(C)
(A)
(B)
### Chart
| Category | |
|---|---|
| MGT (Baseline) | 728.0790000000001 |
| MGT (After 1 week) | 808.0690649999999 |
| EGT (Baseline) | 532.3533333333332 |
| EGT (After 1 week) | 588.3366833333333 |
### Chart
| Category | |
|---|---|
| MGT (Baseline) | 1496.7367499999996 |
| MGT (After 1 week) | 1606.63545 |
| EGT (Baseline) | 1276.2136666666665 |
| EGT (After 1 week) | 1530.3111666666666 |
### Chart
| Category | |
|---|---|
| MGT (Baseline) | 669.21945 |
| MGT (After 1 week) | 750.4837500000001 |
| EGT (Baseline) | 506.3583333333333 |
| EGT (After 1 week) | 544.3998333333334 |(F)
(D)
(E)
### Chart
| Category | |
|---|---|
| MGT (Baseline) | 607.1364 |
| MGT (After 1 week) | 627.4966499999999 |
| EGT (Baseline) | 548.7436666666667 |
| EGT (After 1 week) | 625.9474999999999 |
### Chart
| Category | |
|---|---|
| MGT (Baseline) | 343.64549999999997 |
| MGT (After 1 week) | 362.11935 |
| EGT (Baseline) | 273.75 |
| EGT (After 1 week) | 324.3638333333333 |
### Chart
| Category | |
|---|---|
| MGT (Baseline) | 96.17816666666666 |
| MGT (After 1 week) | 96.75899999999999 |
| EGT (Baseline) | 81.49249999999999 |
| EGT (After 1 week) | 89.06183333333334 |

## Slide 3
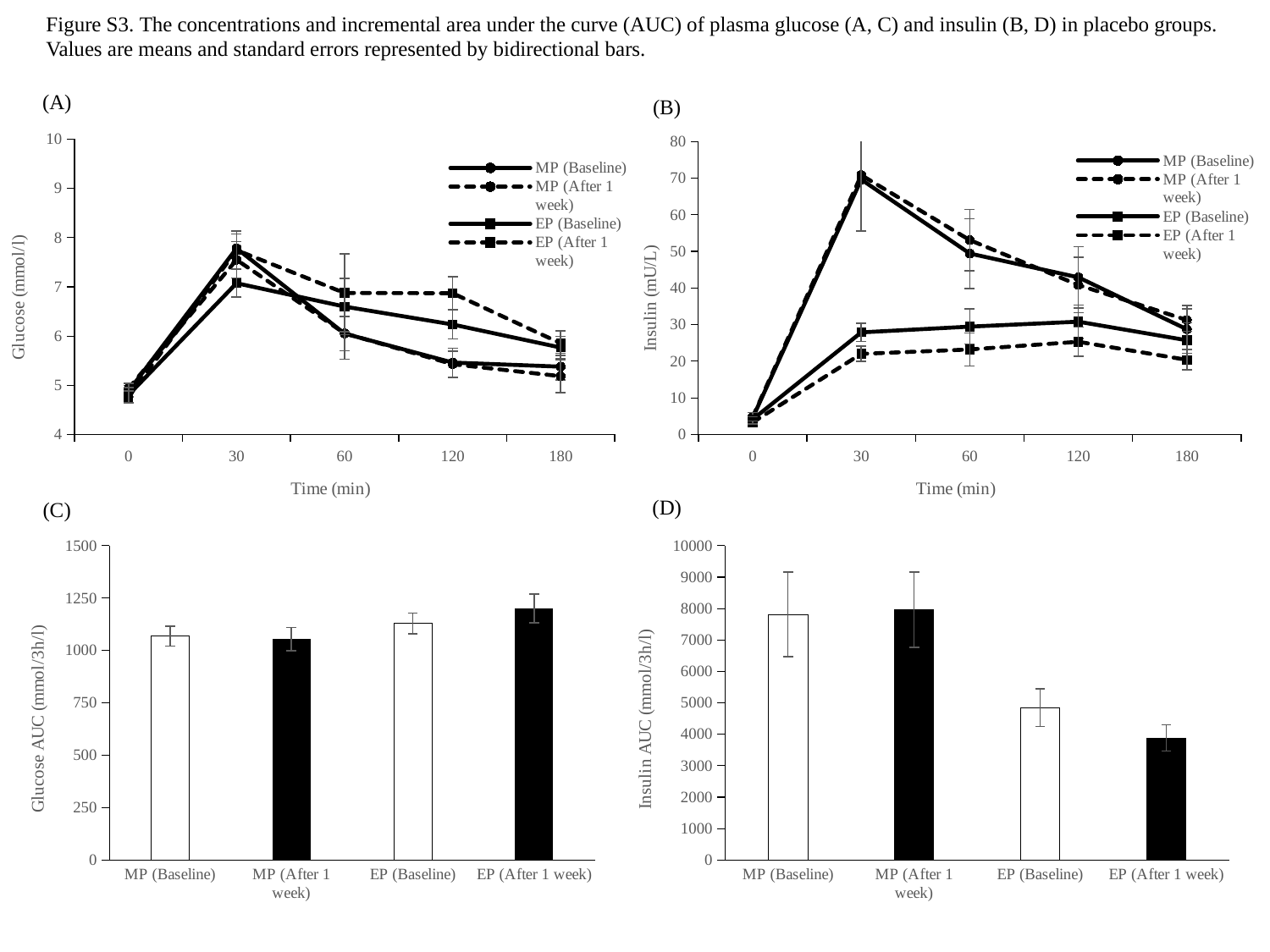

Figure S3. The concentrations and incremental area under the curve (AUC) of plasma glucose (A, C) and insulin (B, D) in placebo groups. Values are means and standard errors represented by bidirectional bars.
(A)
(B)
### Chart
| Category | MP (Baseline) | MP (After 1 week) | EP (Baseline) | EP (After 1 week) |
|---|---|---|---|---|
| 0 | 4.879329 | 4.934838999999999 | 4.804698888888889 | 4.7615244444444444 |
| 30 | 7.782501999999999 | 7.54936 | 7.07444111111111 | 7.746728888888888 |
| 60 | 6.050589999999999 | 6.056140999999999 | 6.599522222222222 | 6.877072222222222 |
| 120 | 5.462184 | 5.428877999999999 | 6.235623333333333 | 6.870904444444444 |
| 180 | 5.378919 | 5.184634 | 5.766872222222222 | 5.853221111111111 |
### Chart
| Category | MP (Baseline) | MP (After 1 week) | EP (Baseline) | EP (After 1 week) |
|---|---|---|---|---|
| 0 | 4.546824427781482 | 4.807268366835068 | 4.138240789921531 | 3.32471343664686 |
| 30 | 69.60270688940015 | 70.80394533505799 | 27.8737918640011 | 22.003211864563763 |
| 60 | 49.39863190097239 | 53.06761736996769 | 29.442346304051654 | 23.209136891631154 |
| 120 | 42.89497467299052 | 40.806892353939546 | 30.79378476972763 | 25.30840772012443 |
| 180 | 28.71865205630047 | 31.23990720845012 | 25.72002595631229 | 20.372058398985878 |(D)
(C)
### Chart
| Category | |
|---|---|
| MP (Baseline) | 1068.040155 |
| MP (After 1 week) | 1054.30143 |
| EP (Baseline) | 1128.4257833333334 |
| EP (After 1 week) | 1201.1438833333332 |
### Chart
| Category | |
|---|---|
| MP (Baseline) | 7814.480050710929 |
| MP (After 1 week) | 7969.8809246926885 |
| EP (Baseline) | 4842.4208163242065 |
| EP (After 1 week) | 3884.0444327870596 |
